# Diagnostic Evaluation of Pulmonary Hypertension: A Comprehensive Approach for Primary Care Physicians

**DOI:** 10.3390/jcm12237309

**Published:** 2023-11-25

**Authors:** Suneesh Anand, Ahmed Sadek, Anjali Vaidya, Estefania Oliveros

**Affiliations:** Department of Medicine, Temple Heart and Vascular Institute, Pulmonary Hypertension, Right Heart Failure, CTEPH Program, Temple University Hospital, Philadelphia, PA 19140, USA; suneesh.anand@tuhs.temple.edu (S.A.); ahmed.sadek@tuhs.temple.edu (A.S.);

**Keywords:** pulmonary hypertension, pulmonary vascular disease, right heart failure

## Abstract

Pulmonary hypertension (PH) is a disorder involving a heterogeneous group of medical conditions encompassing several cardiopulmonary illnesses. Implementing new diagnostic criteria for PH in conjunction with multimodality diagnostic tools is crucial for accurate and early recognition of this life-threatening form of right heart failure. This should streamline early referrals to accredited PH centers, with a goal to rapidly institute targeted therapy in order to optimize prognosis.

## 1. Introduction

Pulmonary hypertension (PH) is a disorder involving a heterogeneous group of medical conditions encompassing several cardiopulmonary illnesses. The most recent 6th World Symposium on Pulmonary Hypertension redefined the threshold for recognizing PH, including a new cut-off level for mean pulmonary artery pressure ≥20 mmHg [1]. The World Health Organization’s diagnostic groups of PH provide a useful framework for categorizing the various etiologies of PH, whereas the hemodynamics more directly allow us to understand the phenotype (i.e., precapillary, postcapillary, or combined pre- and postcapillary).

The diagnosis of PH can be complex, and at times, it requires a multidisciplinary approach in order to detect and manage PH. General practitioners are frequently the first physicians to encounter this group of patients [2]. Despite the life-threatening nature of this condition and the increased awareness and advances in therapies in the past 20 years, significant delays from the onset of symptoms to the time of diagnosis remain. The time from symptom onset to a diagnosis of PH can be delayed by a mean of over 2 years and occur after multiple hospitalizations. The highest likelihood of delayed recognition occurs in patients that are less than 36 years old. Deano et al. [3] demonstrated that 60% of patients had functional class III or IV symptoms, and 33% were misdiagnosed at the time of referral for PH. These delays can result in a worsening clinical outcome or survival [2,3,4,5,6]. We present a practical overview with an emphasis on the early diagnosis of PH for the clinician and suggest pathways for expedited referral to PH centers of excellence to improve outcomes through the early initiation of treatment [7]. As such, the essential aspects in the evaluation and diagnosis of pulmonary hypertension are summarized below. We also include a suggested approach for referral.

## 2. Classification of PH

**Figure 1:**
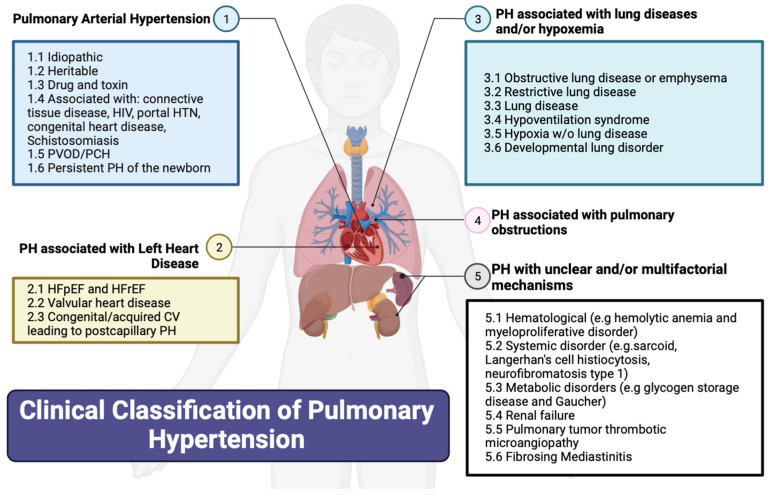
Clinical classification of pulmonary hypertension. CV: cardiovascular; e.g.: example given; HFpEF: heart failure with preserved ejection fraction; HFrEF: heart failure with reduced ejection fraction; HIV: human immunodeficiency virus; HTN: hypertension; PH: pulmonary hypertension; w/o: without. Created using Biorender.

Figure 1 summarizes the updated classification of the 6th World Symposium on PH based on etiology. The WHO’s Group I PH, referred to as pulmonary arterial hypertension (PAH), encompasses various causes including connective tissue diseases (most commonly systemic sclerosis), HIV, portal hypertension, drug and toxin exposures, congenital heart diseases causing systemic-to-pulmonary shunting, and idiopathic and hereditary PH. PAH in particular is an underdiagnosed but serious disease, characterized by progressive right heart failure [8]. The WHO’s Group II PH is PH from left heart diseases, also known as postcapillary or pulmonary venous hypertension and includes left ventricular systolic and/or diastolic heart failure and heart failure related to left-sided valvular disease. The WHO’s Group III PH is caused by chronic hypoxic and respiratory diseases. The WHO’s Group IV PH is related to chronic pulmonary artery obstructions, most commonly chronic thromboembolic pulmonary hypertension (CTEPH). Lastly, the WHO’s Group V PH includes miscellaneous diseases such as sarcoidosis, thyroid disorders, and end-stage renal disease with or without dialysis. 

## 3. History and Physical Exam

By far the most common and one of the earliest presenting symptoms is dyspnea with exertion. This can frequently be the only presenting symptom. The nonspecific nature of this symptom frequently results in misdiagnosis for more common disorders such as asthma, left heart failure, or deconditioning associated with obesity. Orthopnea is more commonly a feature of PH that is secondary to left heart disease as opposed to PAH. Exertional presyncope and syncope are hallmark symptoms of PAH and are frequently what draws attention to the diagnosis. Exertional syncope, as well as rapidly worsening functional capacity, are considered high-risk findings that warrant urgent intervention. Exertional chest pain, which is typically related to right ventricular (RV) ischemia related to limited coronary perfusion in the context of chamber enlargement and hypertrophy, is another common symptom that should be recognized as a manifestation of PAH. Historically, hoarseness (due to compression of the left laryngeal recurrent nerve) and wheezing (due to compression of the bronchi) have been described, but practically, they do not occur, and these symptoms should not be expected or associated with PAH [1,9,10].

During a physical exam, there are multiple findings that can be elicited on cardiac auscultation. Though nonspecific for PH, the systolic murmur of tricuspid regurgitation may be auscultated. An increased pulmonic component to the second heart sound, related to the early closure of the pulmonic valve, may be appreciated. In the setting of RV hypertrophy (RVH) and enlargement, palpation over the sternum may reveal a prominent pulsation, termed the parasternal heave [11]. As the PH syndrome advances, clinical findings of heart failure such as elevated jugular venous pressure, lower extremity edema, and ascites may be apparent. The jugular venous pulsation may have “V” waves, suggesting significant tricuspid regurgitation. The presence of significant right heart failure warrants urgent attention. Resting tachycardia, hypotension, and exertional hypoxia are signs of impaired cardiac output and pulmonary vascular disease and warrant urgent intervention [1,11].

Elements from the patient’s history and physical exam may provide clues to the etiology. A history of Raynaud’s syndrome, dysphagia, or gastroesophageal reflux and physical findings of sclerodactyly or telangiectasias may suggest undiagnosed connective tissue disease. Digital clubbing may suggest a systemic-to-pulmonary shunt with Eisenmenger’s syndrome, which is associated with congenital heart disease or advanced lung disease. An extensive alcohol abuse history or methamphetamine use may suggest Group I PH, related to portopulmonary hypertension or toxins, respectively. Historical elements which predispose patients to left heart disease include traditional cardiovascular risk factors such as the presence of atherosclerosis, systemic arterial hypertension requiring two or more medications, atrial fibrillation, and obesity [12]. An extensive smoking history, abnormal lung sounds, and profound hypoxia may suggest PH in the setting of chronic lung disease. A prior history of hypercoagulable disorders or history of venous thromboembolism may increase suspicion for CTEPH, but the absence of these does not rule out the likelihood of this diagnosis [1,11,13].

## 4. Risk Factors for Pulmonary Hypertension

Certain conditions will be considered risk factors for PH, such as a prior history of pulmonary embolism, use of methamphetamine, connective tissue disease, portal hypertension, HIV, sarcoidosis, congenital heart disease, or a family history of PAH. Screening at-risk patients has been described. In the case of connective-tissue-disease-associated PAH, around 75% have systemic sclerosis, and it carries poor prognosis. Hence, the inclusion of the DETECT algorithm [14] is a Class 1 recommendation in asymptomatic adults with systemic sclerosis of more than 3 years, FVC ≥ 40% and DLCO < 60%.

## 5. Diagnostic Tools

**Figure 2:**
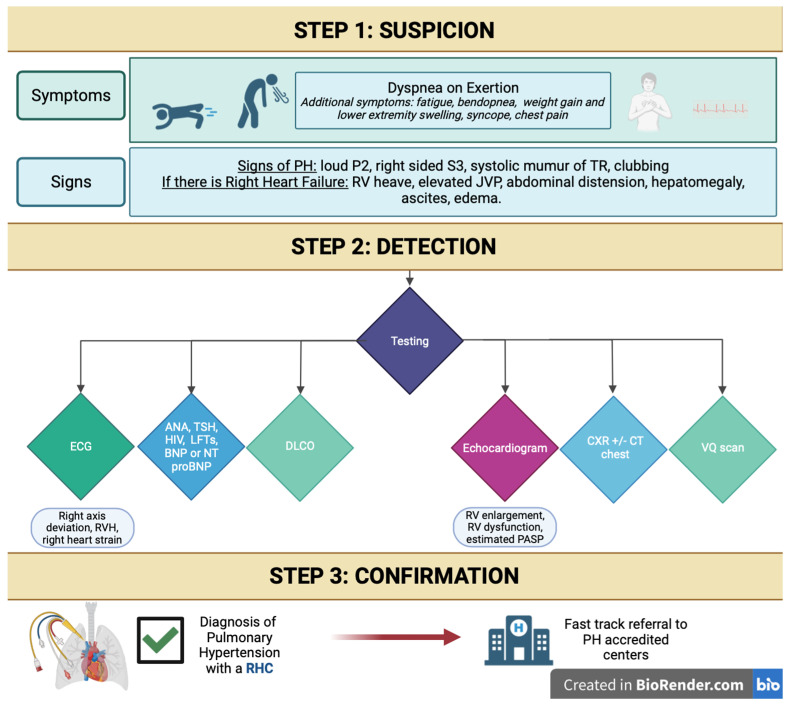
Stepwise approach algorithm for diagnosis of pulmonary hypertension.

We briefly discuss the diagnostic tools available for the diagnosis of PH (Table 1) and summarize our findings in a step-wise algorithmic approach to expedite care for patients with PH (Figure 2).

### 5.1. Laboratory Markers

Laboratory tests that should be obtained at the time of PH diagnosis include blood count, kidney function (creatinine, calculation of estimated glomerular filtration rate, and urea), liver function panel, and BNP or NT-proBNP [1]. Serology testing for HIV and anti-nuclear antibodies should be performed. Screening for biological markers for hypercoagulable diseases is recommended in patients with CTEPH [1].

### 5.2. Electrocardiogram

**Figure 3:**
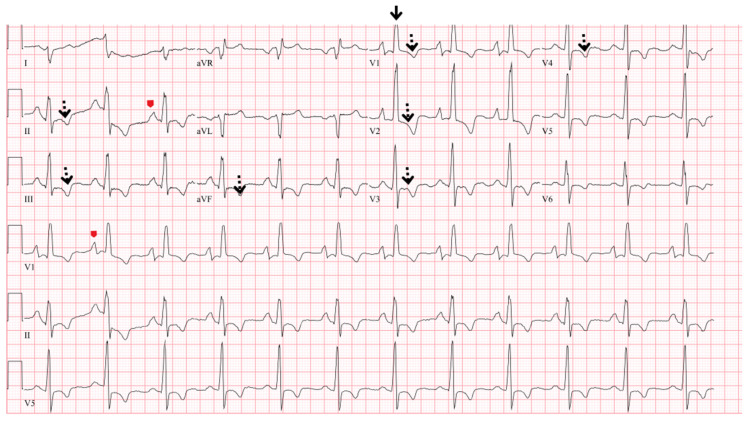
Electrocardiogram findings of pulmonary hypertension. This electrocardiogram demonstrates P pulmonale (arrowheads), right ventricular (RV) hypertrophy (solid arrows), RV strain (dotted arrows), and right axis deviation.

There are typical ECG abnormalities in PH, including P Pulmonale (P wave > 2.5 mm in lead II or prominent positive initial P wave forces in lead V1 or V2), right axis deviation (QRS axis more than 90″ or indeterminate), RVH (R/S > 1, with R > 5 mm in V1; R in V1 + S in lead V5 > 10 mm), right bundle branch block, complete or incomplete (qR or rSR patterns in V1), and RV strain pattern (ST depression/T-wave inversion in the right precordial V1–4 and inferior II, III, aVF leads) [1] (Figure 3). The presence of RAD (right axis deviation) has a high predictive value for PH for patients that are evaluated with unexplained dyspnea on exertion [22]. P wave amplitude in the inferior leads, RVH criteria, and sinus tachycardia have each independently been associated with risk of death [23].

### 5.3. Transthoracic Echocardiography

Transthoracic echocardiography is the first line and the most valuable noninvasive tool in the evaluation of patients with suspected PH. It provides information on right and left heart function, valvular abnormalities, and hemodynamic estimates.

RV systolic pressure (equivalent to pulmonary artery systolic pressure in the absence of congenital pulmonary stenosis) can be estimated based on the peak tricuspid regurgitation velocity (TRV), measured by continuous-wave Doppler (Figure 5A). The echocardiographic estimate for detecting PH is an RV systolic pressure of ≥40 mmHg, which differs from the RHC mean PAP > 20 mmHg. The ESC/ERS guidelines [1] recommend categorizing the probability of detecting PH in three ways based on TRV: low (<2.8 m/s TRV or not measurable), intermediate (2.9–3.4 m/s TRV), and high (>3.4 m/s TRV). They also recommend incorporating RV morphology, size and function, pulmonary artery dimensions, inferior vena cava size, and right atrial area. When used alone in the echocardiographic evaluation of PH, the RV systolic pressure estimate has significant limitations. It does not provide clarity regarding the etiology or diagnostic WHO group of PH and can occasionally be significantly discrepant from invasive hemodynamic measurements [24,25]. Additional limitations include interobserver variability and that it cannot be measured in 20–39% of the patients, particularly in the absence of TR or in patients with obesity or COPD [26].

**Figure 4:**
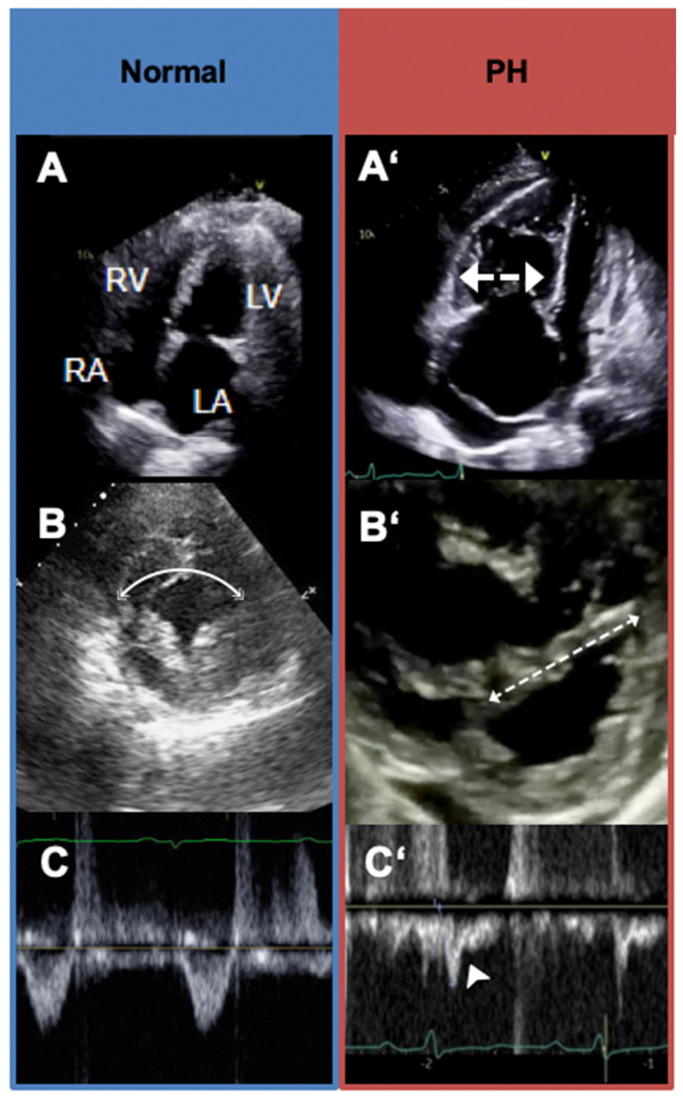
Transthoracic echocardiogram findings of precapillary pulmonary hypertension. In normal physiology, the right ventricle (RV) is approximately 2/3 of the size of the left ventricle (LV); the LV forms the apex of the heart, and the RA is similar in size to the left atrium (**A**). The interventricular septum (IVS) is round ((**B**), line), and the right ventricular outflow tract pulse wave Doppler profile (RVOT PWD) is parabolic (**C**). In pulmonary arterial hypertension, the RV is enlarged and apex-forming ((**A’**), arrow), with right atrial enlargement (**A’**). The IVS can flatten in systole (“pressure“ overload or high resistance) (**B’**). The RVOT PWD is notched in appearance ((**C’**), arrowhead). RA = right atrium, RV = right ventricle, LA = left atrium, LV = left ventricle.

**Figure 5:**
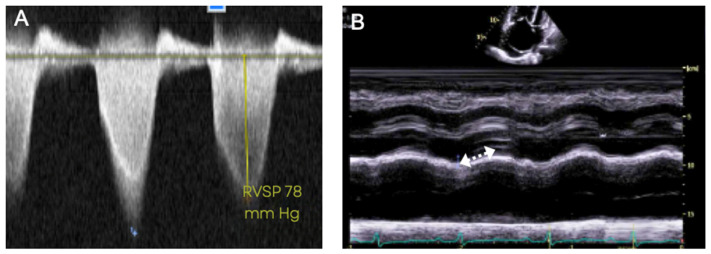
Doppler echocardiographic findings in pulmonary hypertension. The right ventricular systolic pressure (RVSP) can be estimated with the velocity of the tricuspid regurgitant jet by utilizing the modified Bernoulli equation: 4 X (velocity)^2^ + right atrial pressure. In the absence of pulmonic stenosis, the RVSP is equal to the pulmonary artery systolic pressure (**A**). Tricuspid annular plane systolic excursion (TAPSE) is obtained by placing the M-mode cursor in line with the tricuspid annulus. The degree of excursion is utilized to estimate right heart function (**B**). In this patient, the TAPSE is abnormal at 1.0 cm (normal > 1.8 cm).

The features of elevated pulmonary vascular resistance, consistent with WHO Groups 1, 3, and 4 PH, can be recognized on Doppler echocardiography and should raise suspicion for PH even in the presence of a normal RV systolic pressure estimate. A pulse wave Doppler interrogation of the RV outflow tract (RVOT) is performed just proximal to the pulmonic valve. A reduced RVOT Doppler acceleration time (measured as the time from baseline to peak Doppler velocity) or RVOT Doppler systolic notching may be evident (Figure 4C’). Both these Doppler findings are related to the impedance of forward blood flow in the setting of an elevated pulmonary vascular resistance. The position of the interventricular septum is best assessed in short-axis view. In significant pulmonary vascular disease, the interventricular septum will bow abnormally towards the left ventricle during systole, resulting in interventricular septal flattening (Figure 4B’). The RV may be enlarged and hypertrophied with an open apical angle (Figure 4A’). Right heart systolic function, assessed via echocardiogram, is one of the strongest predictors of prognosis in PH. There are various methods to assess right heart function via echocardiography, of which one of the most common is Tricuspid annular plane systolic excursion (TAPSE). This measurement is obtained by placing the M-mode cursor in line with the tricuspid annulus in order to measure the degree of longitudinal excursion (Figure 5) [27,28,29].

Equally important are echocardiographic findings that are more suggestive of WHO Group 2 PH due to left heart disease, which is the most common type of PH. These findings include left atrial enlargement, systolic dysfunction, increased left ventricular mass, significant (grade 2 or worse) diastolic dysfunction, and echo-Doppler estimations of elevated left heart filling pressures [12,30]. The VEST score can be useful as a simple screening tool to assess the likelihood of precapillary (Groups 1, 3, and 4) versus postcapillary PH (Group 2) by extracting common echocardiography parameters from the echocardiographic report. This may help guide timely referral to expert PH centers [30,31]. Echocardiography alone is insufficient to definitively confirm a diagnosis of PH, which requires right heart catheterization (RHC).

### 5.4. Chest X-ray

**Figure 6:**
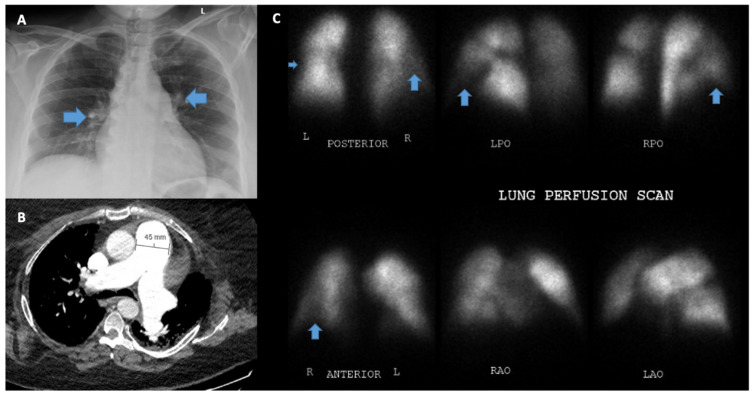
Thoracic imaging of pulmonary hypertension. (**A**) Chest X-ray with prominent pulmonary artery markings. (**B**) CT chest with dilated main pulmonary artery, which is larger than the ascending aorta. (**C**) Perfusion scan with arrows marking abnormal perfusion defects.

A normal chest X-ray does not exclude PH [32]. The characteristic features of PH include a prominent right-sided silhouette due to RA/RV enlargement along with pulmonary artery prominence and pruning of the vessels in the peripheral lung fields [33] (Figure 6). X-ray is useful for differentiating between chronic obstructive airway disease, characterized by enlarged lungs with flattened diaphragms, and restrictive lung disease (interstitial lung diseases), characterized by smaller lung volumes with increased reticular markings. Left heart disease can include left atrial enlargement, interlobular septal thickening, “Kerley B” lines, possible pleural effusions, and the redistribution of pulmonary vessels with upper lobe prominence.

Interestingly, Miniati et al. [20] found that chest radiography has a high sensitivity (96.9%) and specificity (99.1%) for detection of moderate to severe PH. Additionally, chest radiography can show findings of diffuse lung diseases that can be associated with PH, such as interstitial fibrosis and emphysema [10,13].

### 5.5. Pulmonary Function Test

Pulmonary function tests (PFTs) include spirometry, body plethysmography, lung diffusion capacity for carbon monoxide (DLCO), which is useful for evaluating for the presence of chronic lung disease as an etiology for pulmonary hypertension. Spirometry and plethysmography allow for the identification of obstructive or restrictive ventilatory defects. Spirometry in PAH is usually normal or shows at most mild obstructive/restrictive patterns or combined abnormalities. DLCO is usually reduced in PAH, meaning that DLCO (less than 45% of the predicted value) has been recognized in screening patient’s systemic sclerosis for PAH. In this population, a reduced DLCO can be the only abnormality noted on PFTs, and this isolated finding should raise suspicion for the presence of PAH [34]. A low DLCO is associated with a poor prognosis in several forms of PH [35,36,37,38].

### 5.6. Arterial Blood Gas

While arterial blood gas is not required in the diagnosis of PH, patients with PAH have slightly reduced partial pressure of carbon monoxide due to alveolar hyperventilation and normal to slightly reduced partial pressure of arterial oxygen. Elevated PaCO_2_ reflects alveolar hypoventilation and should be evaluated as a possible cause for PH, including the need to do overnight pulse oximetry or polysomnography to evaluate for sleep disorder breathing or hypoventilation. A severely reduced PaO_2_ should raise suspicion for shunting, such as in patent foramen ovale and hepatic disease [39].

### 5.7. Computed Tomography of the Chest and Pulmonary Angiography

Noncontrast computed tomography (CT) of the chest provides important information for evaluating patients with suspected or confirmed PH for features of parenchymal lung disease. A high-resolution CT chest helps to further identify characteristic morphological patterns to diagnose specific clinical entities of interstitial lung disease. The CT signs suggesting the presence of PH include an enlarged PA diameter, a PA-to-aorta ratio >0.9, and enlarged right heart chambers [40]. However, an enlarged pulmonary artery diameter does not exclude PH due to its poor negative predictive value. An enlarged PA diameter can be observed in ILD patients and lung transplant patients without PH [41].

CT imaging can reveal a mosaic attenuation pattern in the lung parenchyma, characterized by areas of hyperperfused vascular segments, intermingled with areas of low attenuation with hypoperfused vascular segments [42]. Amongst the different PH etiologies, PAH and CTEPH are the ones to most commonly show a mosaic pattern on CT. In PAH, mosaicism often manifests as small, scattered areas of increased attenuation, often confined to center of the secondary pulmonary lobule or centrilobular pattern (2). Compared with the mosaic pattern seen in PAH, the pattern in CTEPH often manifests as larger, regional areas of decreased attenuation that correspond to a vascular territory, with associated narrowing or occlusion of the supplying vessel due to the presence of chronic thromboembolic material [43]. CT scans in patients with pulmonary venous hypertension show pulmonary interstitial and alveolar edema.

A CT pulmonary angiography can be used to detect signs of CTEPH such as chronic thromboembolic disease with intravascular webs, bands, occlusions, poststenotic dilatations, and systemic-to-pulmonary arterial collateral vessels. Catheter-directed digital subtraction angiography (DSA) with conventional two-planar imaging should only be performed at expert CTEPH centers to further the diagnostic assessment of CTEPH, including to assess candidacy for pulmonary thromboendarterectomy or balloon pulmonary angioplasty [10,13].

### 5.8. Ventilation/Perfusion Scanning (V/Q)

It is strongly recommended that all patients with precapillary PH undergo testing to exclude CTEPH. The clinical presentation of patients with Group I and Group IV PH can, otherwise, be very similar. A VQ scan is the gold standard screening test to evaluate for CTEPH, as a normal perfusion scan excludes CTEPH with a negative predictive value of 98% [22]. In patients with PAH, VQ is typically normal but may occasionally show a speckled, heterogeneous pattern, a pattern that is not consistent with chronic thromboembolic disease [44,45]. Matched defects in patients with PH are more consistent with Group 3 PH with parenchymal lung abnormalities [1].

### 5.9. Cardiac Magnetic Resonance Imaging

Cardiovascular magnetic resonance (CMR) has a unique capability in providing an accurate and reproducible assessment of cardiac function, disease severity, and tissue characterization. It has emerged over the last decade but remains primarily used for complex assessment of congenital-heart-disease-associated pulmonary hypertension. The key indicators of PH can be retrieved, such as main pulmonary artery dilation, quantification of RV parameters (i.e., volume, ejection fraction, mass, and septal angle) and hemodynamics (i.e., stroke volume index), and the presence of a dilated right atrium or flattened or reversed septum curvature with dyskinetic motion; this can also be assessed via phase-contrast MRI with diagnostic precision [46]. CMR can help with distinguishing the PH subtype by providing information of left heart disease (Group 2) [47]. There is a paucity of data on the different PH subtypes, which is a limitation for the generalizability of CMR-derived parameters. In addition, the cost and availability of the technique is another limitation for general implementation of this imaging modality.

### 5.10. Right Heart Catheterization

RHC is the gold standard for hemodynamically classifying PH and guiding therapy [1]. It is a relatively low-risk procedure with serious adverse events accounting for 1.1% and low procedure-related mortality (0.055%) when performed at PH centers [48]. RHC provides relevant data, including right- and left-sided filling pressures, pulmonary arterial pressure (PAP), pulmonary arterial wedge pressure (PAWP), which is a surrogate for left atrial pressure, pulmonary vascular resistance (PVR), cardiac output (CO), and cardiac index (CI) [49,50]. CO should be assessed by the direct Fick or thermodilution (mean values of at least three measurements) methods.

PH can be grouped phenotypically into precapillary, postcapillary, or combined pre- and postcapillary PH. Precapillary PH (WHO Groups I, III, IV, and V PH) is defined by an elevated mean pulmonary artery pressure (MPAP), with a PAWP ≤ 15 mm Hg, and an elevated PVR. Postcapillary PH (WHO Group II PH) is defined by an elevated MPAP, PAWP > 15 mm Hg, and normal PVR. Combined pre- and postcapillary PH is defined by an elevated MPAP and both PAWP > 15 mm Hg and elevated PVR. Patients with significant precapillary pulmonary hypertension should be referred for evaluation of PH-directed therapy, as should select cases of combined pre- and postcapillary PH. Classically, PVR ≥ 3 Wood units (WU) with an MPAP > 25 mm Hg has been considered abnormal. However, the 2022 ESC/ERS PH guidelines have lowered the threshold of abnormal MPAP to greater than 20 mmHg and PVR to greater than 2 WU based on studies suggesting adverse outcomes at this lower threshold in various disease states [1,51,52,53]. This can prompt even earlier recognition and referral to expert PH centers. Nonetheless, a PVR between 2 and 3 WU with an MPAP between 20 and 25 constitutes a less clear therapeutic target in PH-directed therapy, as landmark PH drug trials predominantly included patients with higher PVR and MPAP [54,55,56]. PH-directed therapy in this group should be assessed on an individual basis at expert PH centers.

Great care should be taken to ensure proper acquisition of hemodynamic measurements. The external pressure transducer should be zeroed at the level of the left atrium with the patient lying supine. All measurements, including PAWP, should be measured at the end expiration (without breath-holding maneuver). Of all the hemodynamic measurements, acquisition of the PAWP is the most susceptible to technical errors, especially in those with precapillary PH due to increased caliber and stiffness of the pulmonary arteries, leading to an increased chance of “under-occluding” the vessel and thus falsely over-estimating the PAWP. This error can lead to PH misclassification as WHO Group 2 PH and have profound negative implications for delayed diagnosis and implementation of appropriate medical therapy. RHC measurements should not be interpreted in isolation and should be scrutinized against other available data, in particular the echocardiogram, to ensure concordance with the overall clinical picture [57].

### 5.11. Cardiopulmonary Exercise Testing

Cardiopulmonary exercise testing (CPET) is an invaluable tool in the evaluation of unexplained dyspnea on exertion and the integrative exercise responses of different organ systems. Patients with PAH show a typical pattern, with a low end-tidal partial pressure of carbon dioxide (PET_CO2_), high ventilatory equivalent for carbon dioxide (V_E_/V_CO2_), low oxygen pulse (V_O2_/HR), and low peak oxygen uptake (V_O2_) [58]. While not routinely required for the diagnosis of PH, it can be helpful in complex cases of dyspnea in which PH is a possible contributing factor. These should be performed at expert centers with advanced PH expertise. The latest ESC/ERS guidelines [1] have also incorporated their use for consideration during risk stratification of PAH, although they are not routinely performed for this purpose.

## 6. Suggested Initial Evaluation by the Primary Care Provider for Suspected PH and Indications for Referral to Expert PH Center

Patients that are suspected to have PH or those with unexplained dyspnea are commonly seen by primary care physicians. The initial work-up starts with obtaining a detailed medical history and physical examination. The initial diagnostic tests can easily be obtained in the office or with routine lab work and should ideally include ambulatory oxygen saturation, BNP or NT-pro BNP, and ECG. As a next step, chest X-ray, pulmonary function testing, and echocardiography are easily available noninvasive tests that can be obtained by the primary care practitioner. These tests are not only useful in the evaluation of PH, but as a general assessment of other common cardiac and pulmonary etiologies of unexplained dyspnea. An echocardiogram is crucial in helping to identify the probability of PH, irrespective of the cause. One of the challenges with PH is that a definitive diagnosis needs to be made with an RHC. Therefore, it is important to consider the pretest probability of treatable PAH. If routine examinations indicate an alternative, then diagnosis of PAH or CTEPH should not be pursued.

As previously discussed, a tricuspid regurgitant velocity (TRV) of less than 2.9 m/s, in the absence of other signs of elevated pulmonary vascular resistance (Figure 4 and Figure 5) or clinical risk factors for PH such as connective tissue disease, suggests a low probability of PH. In such patients, an alternative cause of dyspnea should be pursued. Patients with a TRV of 2.9–3.4 m/s (intermediate probability) without echocardiographic signs of right ventricular enlargement or dysfunction, elevated pulmonary vascular resistance, or clinical risk factors for PH, may benefit from evaluation by a general cardiologist but may not necessarily require referral to a PH specialist. For Group 2 PH patients with a TRV greater than 3.4 m/s in the context of clear echocardiographic findings, normal right heart size and function, and none of the findings of elevated pulmonary vascular resistance, patients are unlikely to have significant precapillary PH. These patients can benefit from a cardiology referral for management of left heart disease, but may not need referral to a PH specialist. Patients with echocardiographic signs of elevated pulmonary vascular resistance should be referred to a PH specialist regardless of their RV systolic pressure, estimated via TRV. In these patients, a normal estimated RV systolic pressure is unlikely to be accurate [25]. Referrals to PH centers should take place in cases of intermediate/high probability of PH, risk factors for PAH, and concerns of CTEPH. In this particular subset of patients, obtaining a comprehensive laboratory evaluation for possible etiologies of PH as well as a ventilation–perfusion scan for CTEPH evaluation can be obtained simultaneously with PH specialist referral in order to expedite the process. In fact, a VQ scan should ideally be obtained by the primary care practitioner in any patient with a prior history of venous thromboembolism and unexplained dyspnea, even in the absence of echocardiographic signs of PH. Right heart catheterization and cardiac MRI can be reserved to be performed at the PH center as the clinical situation entails.

High-risk PH findings should be recognized and should prompt more urgent referral. These include syncope, rapidly worsening functional capacity (WHO-FC III/IV), the presence of significant right heart failure, RV dysfunction assessed via echocardiography, and signs of hemodynamic instability (i.e., low cardiac output, hypotension, tachycardia).

Patients with scleroderma spectrum connective tissue disease warrant special care due to the high prevalence of PAH and its aggressive nature in this patient group. In these patients, screening for the risk of PAH and the need for referral may be guided by algorithms such as DETECT [14], which utilizes noninvasive testing that is available to the primary care practitioner. It would be reasonable to refer patients in this subgroup with continued dyspnea that is not explained by the results of noninvasive testing as above to a PH specialist for further evaluation.

## 7. Conclusions

Implementing new diagnostic criteria for PH in conjunction with multimodality diagnostic tools is crucial for the accurate and early recognition of this life-threatening form of right heart failure. This should streamline early referrals to accredited PH centers with a goal of rapidly instituting targeted therapy to optimize the prognosis.

## Figures and Tables

**Table 1:** Testing to diagnose pulmonary hypertension.

Tests	Sensitivity	Specificity	Benefit	Limitation
ECG	20% [15]	79.3–100% [16]	Easy to obtain, provides important clues to PH when symptoms are present, helps detect arrhythmia.	ECG considered inadequate for screening.
TTE [17]	83%	72%	Useful initial noninvasive modality for screening and measurement of pulmonary pressures.	Dependence on the quality of imaging, difficulty in image acquisition with increased RV volumes, steady heart rate, and experience of the laboratory staff.
CT chest [18]	74–79%	81–83%	CT chest allows for comprehensive evaluation of the pulmonary vasculature and lung parenchyma.	Radiation exposure.
VQ scan [19,20]	90–100%	94–100%	Allows us to distinguish CTEPH from other forms of PH, negative test helpful for ruling out CTEPH.	Low utility in diagnosing causes of PH other than thromboembolic disease.
CMR [21]	84%	71%	Provides a comprehensive evaluation of the heart, good for quantification of right ventricular volumes, mass and function.	CMR is expensive, not widely available, and requires significant operator expertise.Also limited lung parenchyma evaluation.

## Data Availability

Not applicable.
